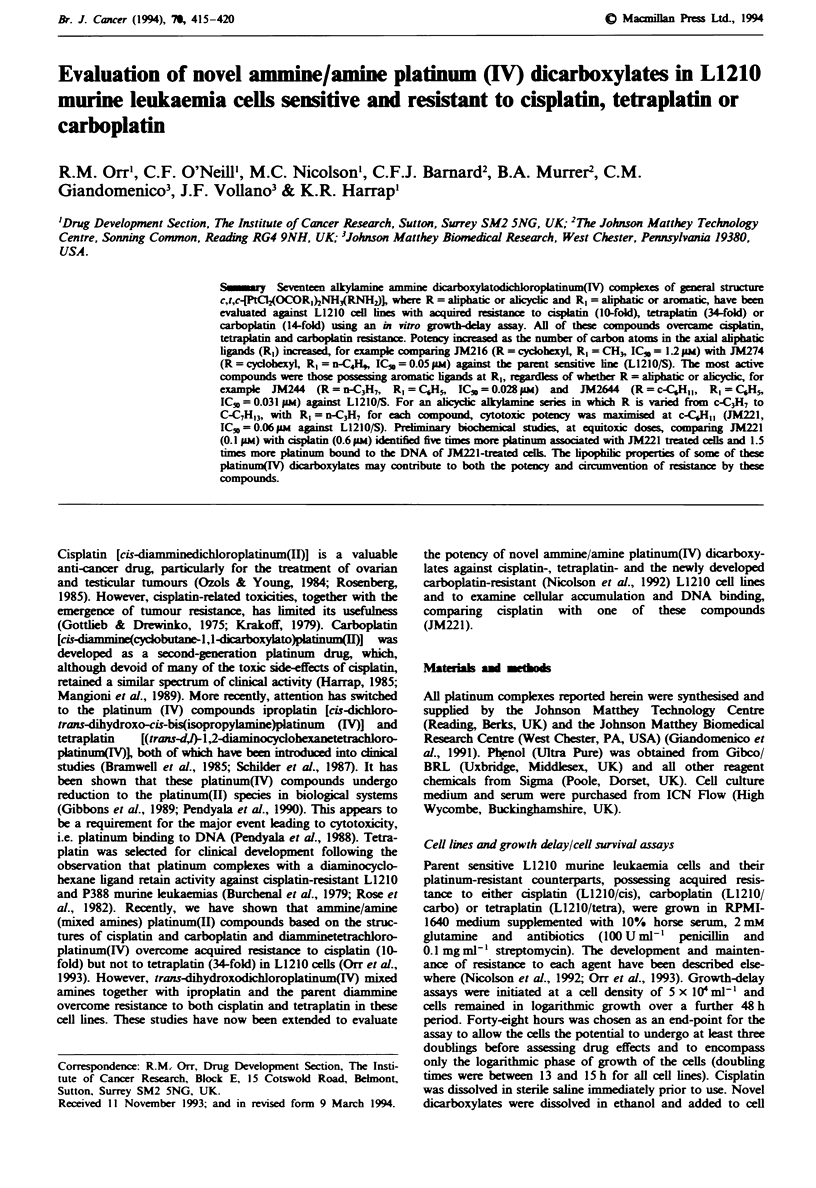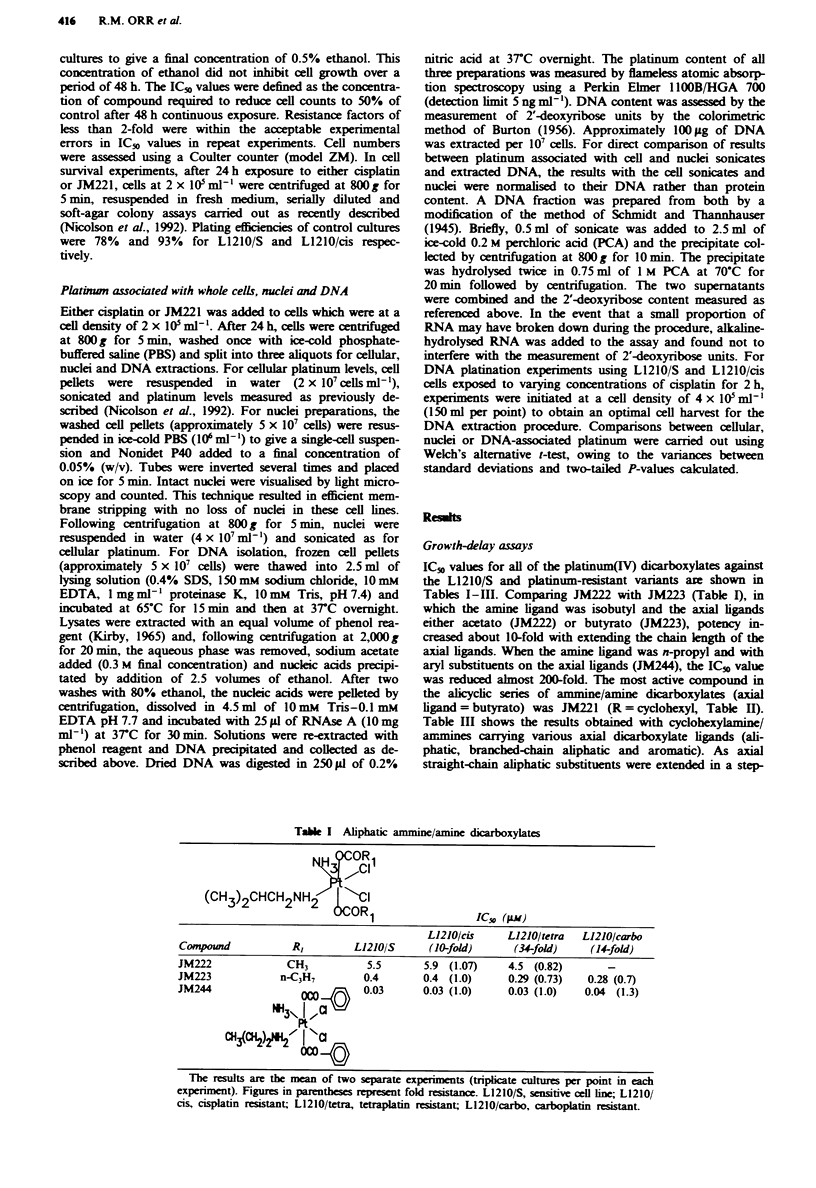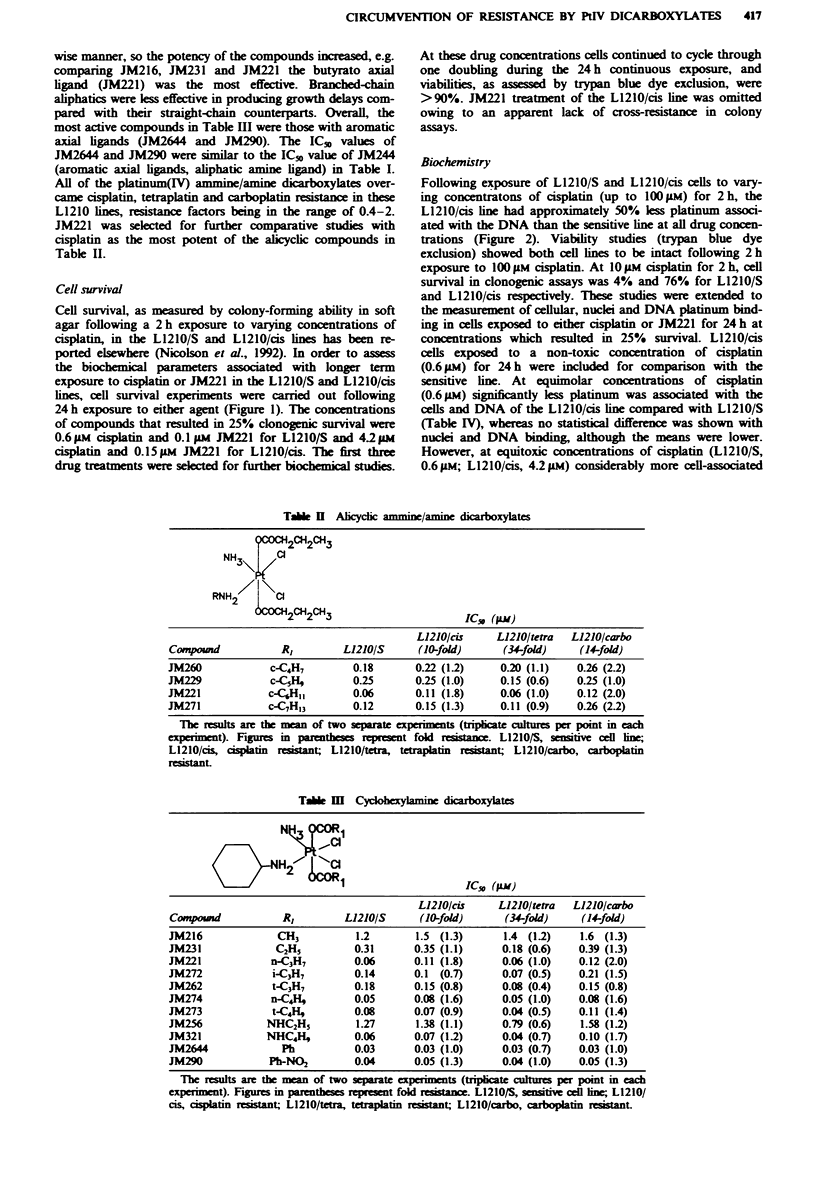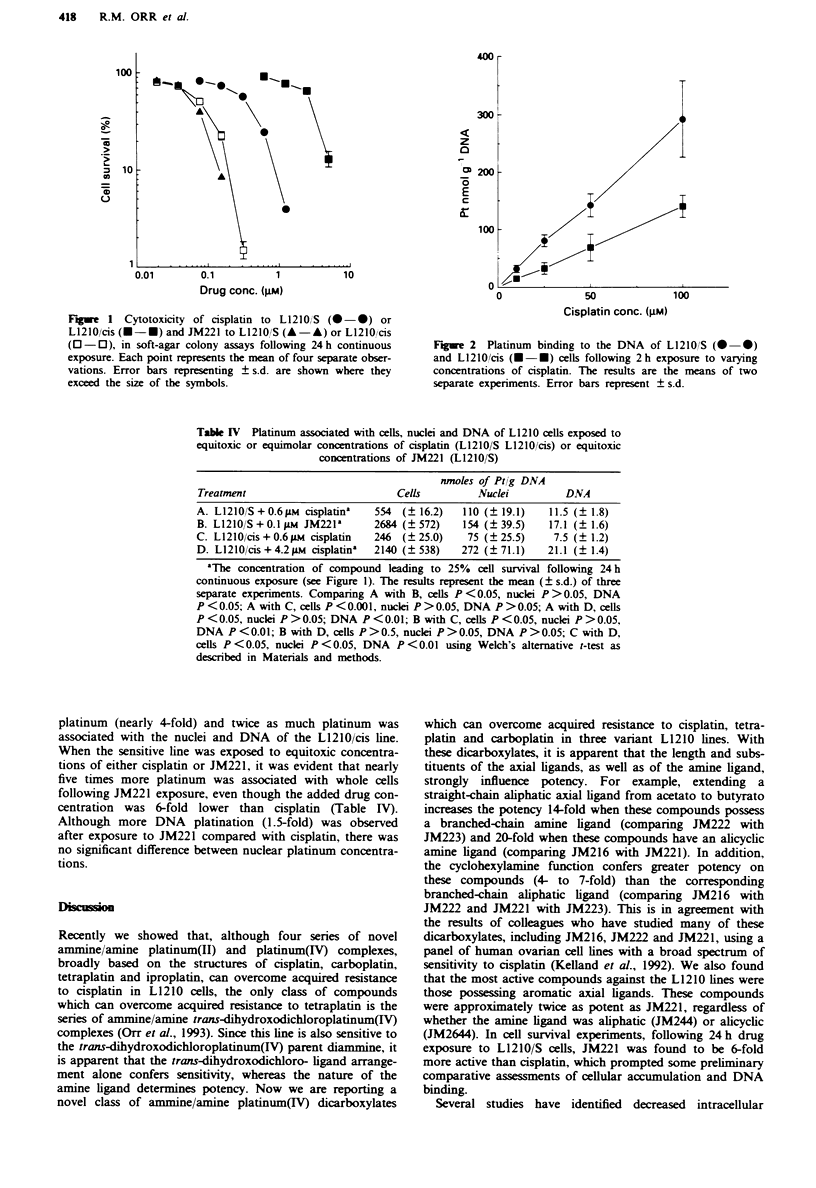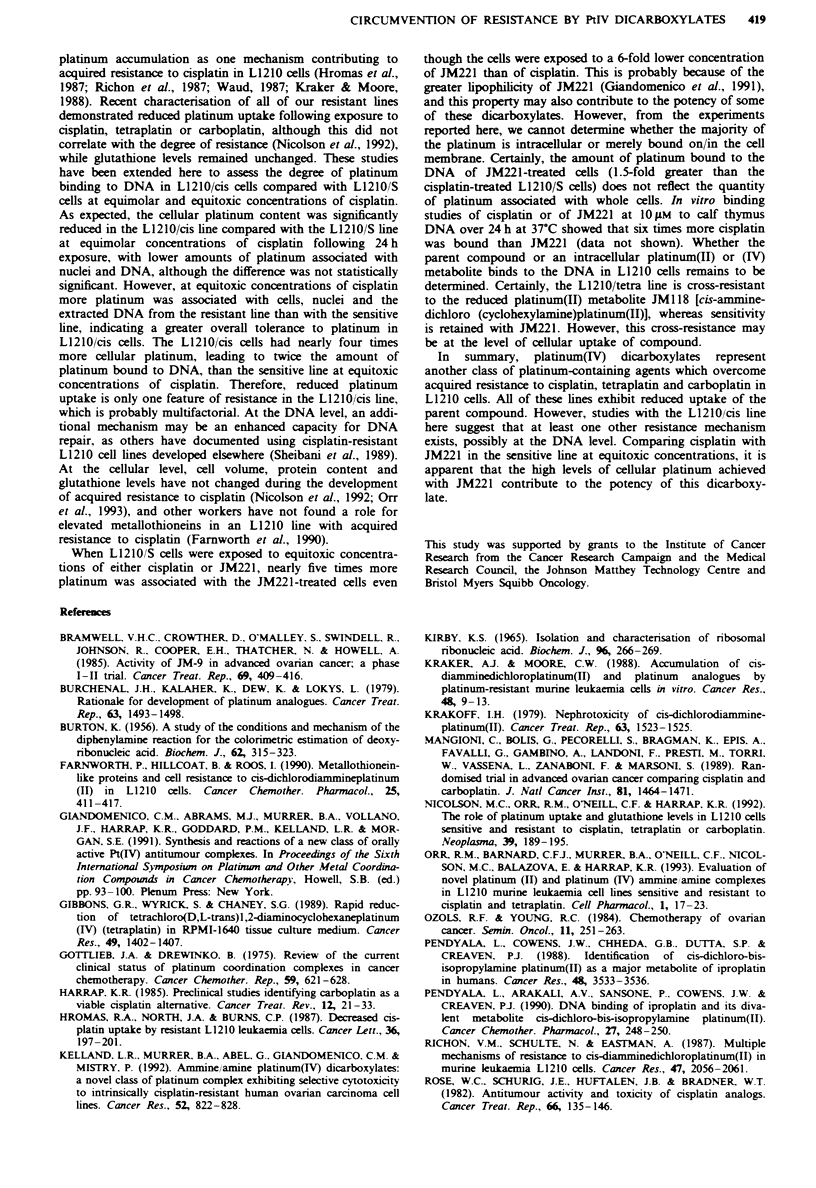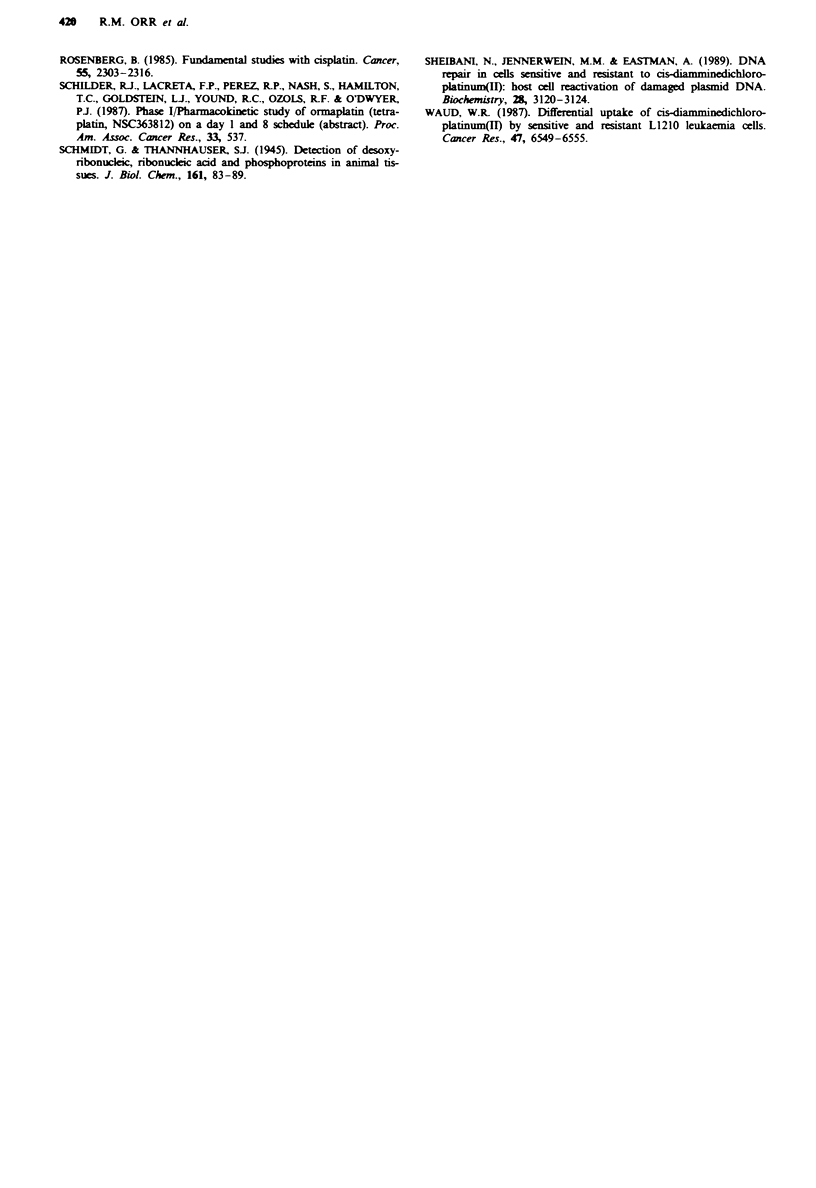# Evaluation of novel ammine/amine platinum (IV) dicarboxylates in L1210 murine leukaemia cells sensitive and resistant to cisplatin, tetraplatin or carboplatin.

**DOI:** 10.1038/bjc.1994.320

**Published:** 1994-09

**Authors:** R. M. Orr, C. F. O'Neill, M. C. Nicolson, C. F. Barnard, B. A. Murrer, C. M. Giandomenico, J. F. Vollano, K. R. Harrap

**Affiliations:** Drug Development Section, Institute of Cancer Research, Sutton, Surrey, UK.

## Abstract

Seventeen alkylamine ammine dicarboxylatodichloroplatinum(IV) complexes of general structure c,t,c-[PtCl2(OCOR1)2NH3(RNH2)], where R = aliphatic or alicyclic and R1 = aliphatic or aromatic, have been evaluated against L1210 cell lines with acquired resistance to cisplatin (10-fold), tetraplatin (34-fold) or carboplatin (14-fold) using an in vitro growth-delay assay. All of these compounds overcame cisplatin, tetraplatin and carboplatin resistance. Potency increased as the number of carbon atoms in the axial aliphatic ligands (R1) increased, for example comparing JM216 (R = cyclohexyl, R1 = CH3, IC50 = 1.2 microM) with JM274 (R = cyclohexyl, R1 = n-C4H9, IC50 = 0.05 microM) against the parent sensitive line (L1210/S). The most active compounds were those possessing aromatic ligands at R1, regardless of whether R = aliphatic or alicyclic, for example JM244 (R = n-C3H7, R1 = C6H5, IC50 = 0.028 microM) and JM2644 (R = c-C6H11, R1 = C6H5, IC50 = 0.031 microM) against L1210/S. For an alicyclic alkylamine series in which R is varied from c-C3H7 to C-C7H13, with R1 = n-C3H7 for each compound, cytotoxic potency was maximised at c-C6H11 (JM221, IC50 = 0.06 microM against L1210/S). Preliminary biochemical studies, at equitoxic doses, comparing JM221 (0.1 microM) with cisplatin (0.6 microM) identified five times more platinum associated with JM221 treated cells and 1.5 times more platinum bound to the DNA of JM221-treated cells. The lipophilic properties of some of these platinum(IV) dicarboxylates may contribute to both the potency and circumvention of resistance by these compounds.


Br. J. Cancer (1994), 70, 415–420

© Macmillan Press Ltd., 1994

### Evaluation of novel ammine/amine platinum (IV) dicarboxylates in L1210 murine leukaemia cells sensitive and resistant to cisplatin, tetraplatin or carboplatin

R.M. Orr[1], C.F. O'Neill[1], M.C. Nicolson[1], C.F.J. Barnard[2], B.A. Murrer[2], C.M. Giandomenico[3], J.F. Vollano[3] & K.R. Harrap[1]

[1]*Drug Development Section, The Institute of Cancer Research, Sutton, Surrey SM2 5NG, UK;* [2]*The Johnson Matthey Technology Centre, Sonning Common, Reading RG4 9NH, UK;* [3]*Johnson Matthey Biomedical Research, West Chester, Pennsylvania 19380, USA.*

**Summary** Seventeen alkylamine ammine dicarboxylatodichloroplatinum(IV) complexes of general structure *c,t,c*-$[PtCl_2(OCOR_1)_2NH_3(RNH_2)]$, where R = aliphatic or alicyclic and $R_1$ = aliphatic or aromatic, have been evaluated against L1210 cell lines with acquired resistance to cisplatin (10-fold), tetraplatin (34-fold) or carboplatin (14-fold) using an *in vitro* growth-delay assay. All of these compounds overcame cisplatin, tetraplatin and carboplatin resistance. Potency increased as the number of carbon atoms in the axial aliphatic ligands ($R_1$) increased, for example comparing JM216 (R = cyclohexyl, $R_1 = CH_3$, $IC_{50} = 1.2$ μM) with JM274 (R = cyclohexyl, $R_1$ = n-$C_4H_9$, $IC_{50} = 0.05$ μM) against the parent sensitive line (L1210/S). The most active compounds were those possessing aromatic ligands at $R_1$, regardless of whether R = aliphatic or alicyclic, for example JM244 (R = n-$C_3H_7$, $R_1 = C_6H_5$, $IC_{50} = 0.028$ μM) and JM2644 (R = c-$C_6H_{11}$, $R_1 = C_6H_5$, $IC_{50} = 0.031$ μM) against L1210/S. For an alicyclic alkylamine series in which R is varied from c-$C_3H_7$ to C-$C_7H_{13}$, with $R_1$ = n-$C_3H_7$ for each compound, cytotoxic potency was maximised at c-$C_6H_{11}$ (JM221, $IC_{50} = 0.06$ μM against L1210/S). Preliminary biochemical studies, at equitoxic doses, comparing JM221 (0.1 μM) with cisplatin (0.6 μM) identified five times more platinum associated with JM221 treated cells and 1.5 times more platinum bound to the DNA of JM221-treated cells. The lipophilic properties of some of these platinum(IV) dicarboxylates may contribute to both the potency and circumvention of resistance by these compounds.

Cisplatin [*cis*-diamminedichloroplatinum(II)] is a valuable anti-cancer drug, particularly for the treatment of ovarian and testicular tumours (Ozols & Young, 1984; Rosenberg, 1985). However, cisplatin-related toxicities, together with the emergence of tumour resistance, has limited its usefulness (Gottlieb & Drewinko, 1975; Krakoff, 1979). Carboplatin [*cis*-diammine(cyclobutane-1,1-dicarboxylato)platinum(II)] was developed as a second-generation platinum drug, which, although devoid of many of the toxic side-effects of cisplatin, retained a similar spectrum of clinical activity (Harrap, 1985; Mangioni *et al.*, 1989). More recently, attention has switched to the platinum (IV) compounds iproplatin [*cis*-dichloro-*trans*-dihydroxo-*cis*-bis(isopropylamine)platinum (IV)] and tetraplatin [(*trans*-*d,l*)-1,2-diaminocyclohexanetetrachloroplatinum(IV)], both of which have been introduced into clinical studies (Bramwell *et al.*, 1985; Schilder *et al.*, 1987). It has been shown that these platinum(IV) compounds undergo reduction to the platinum(II) species in biological systems (Gibbons *et al.*, 1989; Pendyala *et al.*, 1990). This appears to be a requirement for the major event leading to cytotoxicity, i.e. platinum binding to DNA (Pendyala *et al.*, 1988). Tetraplatin was selected for clinical development following the observation that platinum complexes with a diaminocyclohexane ligand retain activity against cisplatin-resistant L1210 and P388 murine leukaemias (Burchenal *et al.*, 1979; Rose *et al.*, 1982). Recently, we have shown that ammine/amine (mixed amines) platinum(II) compounds based on the structures of cisplatin and carboplatin and diamminetetrachloroplatinum(IV) overcome acquired resistance to cisplatin (10-fold) but not to tetraplatin (34-fold) in L1210 cells (Orr *et al.*, 1993). However, *trans*-dihydroxodichloroplatinum(IV) mixed amines together with iproplatin and the parent diammine overcome resistance to both cisplatin and tetraplatin in these cell lines. These studies have now been extended to evaluate the potency of novel ammine/amine platinum(IV) dicarboxylates against cisplatin-, tetraplatin- and the newly developed carboplatin-resistant (Nicolson *et al.*, 1992) L1210 cell lines and to examine cellular accumulation and DNA binding, comparing cisplatin with one of these compounds (JM221).

Correspondence: R.M. Orr, Drug Development Section, The Institute of Cancer Research, Block E, 15 Cotswold Road, Belmont, Sutton, Surrey SM2 5NG, UK.
Received 11 November 1993; and in revised form 9 March 1994.

### Materials and methods

All platinum complexes reported herein were synthesised and supplied by the Johnson Matthey Technology Centre (Reading, Berks, UK) and the Johnson Matthey Biomedical Research Centre (West Chester, PA, USA) (Giandomenico *et al.*, 1991). Phenol (Ultra Pure) was obtained from Gibco/BRL (Uxbridge, Middlesex, UK) and all other reagent chemicals from Sigma (Poole, Dorset, UK). Cell culture medium and serum were purchased from ICN Flow (High Wycombe, Buckinghamshire, UK).

### *Cell lines and growth delay/cell survival assays*

Parent sensitive L1210 murine leukaemia cells and their platinum-resistant counterparts, possessing acquired resistance to either cisplatin (L1210/cis), carboplatin (L1210/carbo) or tetraplatin (L1210/tetra), were grown in RPMI-1640 medium supplemented with 10% horse serum, 2 mM glutamine and antibiotics (100 U $ml^{-1}$ penicillin and 0.1 mg $ml^{-1}$ streptomycin). The development and maintenance of resistance to each agent have been described elsewhere (Nicolson *et al.*, 1992; Orr *et al.*, 1993). Growth-delay assays were initiated at a cell density of $5 \times 10^4$ $ml^{-1}$ and cells remained in logarithmic growth over a further 48 h period. Forty-eight hours was chosen as an end-point for the assay to allow the cells the potential to undergo at least three doublings before assessing drug effects and to encompass only the logarithmic phase of growth of the cells (doubling times were between 13 and 15 h for all cell lines). Cisplatin was dissolved in sterile saline immediately prior to use. Novel dicarboxylates were dissolved in ethanol and added to cell cultures to give a final concentration of 0.5% ethanol. This concentration of ethanol did not inhibit cell growth over a period of 48 h. The $IC_{50}$ values were defined as the concentration of compound required to reduce cell counts to 50% of control after 48 h continuous exposure. Resistance factors of less than 2-fold were within the acceptable experimental errors in $IC_{50}$ values in repeat experiments. Cell numbers were assessed using a Coulter counter (model ZM). In cell survival experiments, after 24 h exposure to either cisplatin or JM221, cells at $2 \times 10^5\ ml^{-1}$ were centrifuged at 800 g for 5 min, resuspended in fresh medium, serially diluted and soft-agar colony assays carried out as recently described (Nicolson *et al.*, 1992). Plating efficiencies of control cultures were 78% and 93% for L1210/S and L1210/cis respectively.

### *Platinum associated with whole cells, nuclei and DNA*

Either cisplatin or JM221 was added to cells which were at a cell density of $2 \times 10^5\ ml^{-1}$. After 24 h, cells were centrifuged at 800 g for 5 min, washed once with ice-cold phosphate-buffered saline (PBS) and split into three aliquots for cellular, nuclei and DNA extractions. For cellular platinum levels, cell pellets were resuspended in water ($2 \times 10^7$ cells $ml^{-1}$), sonicated and platinum levels measured as previously described (Nicolson *et al.*, 1992). For nuclei preparations, the washed cell pellets (approximately $5 \times 10^7$ cells) were resuspended in ice-cold PBS ($10^6\ ml^{-1}$) to give a single-cell suspension and Nonidet P40 added to a final concentration of 0.05% (w/v). Tubes were inverted several times and placed on ice for 5 min. Intact nuclei were visualised by light microscopy and counted. This technique resulted in efficient membrane stripping with no loss of nuclei in these cell lines. Following centrifugation at 800 g for 5 min, nuclei were resuspended in water ($4 \times 10^7\ ml^{-1}$) and sonicated as for cellular platinum. For DNA isolation, frozen cell pellets (approximately $5 \times 10^7$ cells) were thawed into 2.5 ml of lysing solution (0.4% SDS, 150 mM sodium chloride, 10 mM EDTA, 1 mg $ml^{-1}$ proteinase K, 10 mM Tris, pH 7.4) and incubated at 65°C for 15 min and then at 37°C overnight. Lysates were extracted with an equal volume of phenol reagent (Kirby, 1965) and, following centrifugation at 2,000 g for 20 min, the aqueous phase was removed, sodium acetate added (0.3 M final concentration) and nucleic acids precipitated by addition of 2.5 volumes of ethanol. After two washes with 80% ethanol, the nucleic acids were pelleted by centrifugation, dissolved in 4.5 ml of 10 mM Tris–0.1 mM EDTA pH 7.7 and incubated with 25 µl of RNAse A (10 mg $ml^{-1}$) at 37°C for 30 min. Solutions were re-extracted with phenol reagent and DNA precipitated and collected as described above. Dried DNA was digested in 250 µl of 0.2% nitric acid at 37°C overnight. The platinum content of all three preparations was measured by flameless atomic absorption spectroscopy using a Perkin Elmer 1100B/HGA 700 (detection limit 5 ng $ml^{-1}$). DNA content was assessed by the measurement of 2'-deoxyribose units by the colorimetric method of Burton (1956). Approximately 100 µg of DNA was extracted per $10^7$ cells. For direct comparison of results between platinum associated with cell and nuclei sonicates and extracted DNA, the results with the cell sonicates and nuclei were normalised to their DNA rather than protein content. A DNA fraction was prepared from both by a modification of the method of Schmidt and Thannhauser (1945). Briefly, 0.5 ml of sonicate was added to 2.5 ml of ice-cold 0.2 M perchloric acid (PCA) and the precipitate collected by centrifugation at 800 g for 10 min. The precipitate was hydrolysed twice in 0.75 ml of 1 M PCA at 70°C for 20 min followed by centrifugation. The two supernatants were combined and the 2'-deoxyribose content measured as referenced above. In the event that a small proportion of RNA may have broken down during the procedure, alkaline-hydrolysed RNA was added to the assay and found not to interfere with the measurement of 2'-deoxyribose units. For DNA platination experiments using L1210/S and L1210/cis cells exposed to varying concentrations of cisplatin for 2 h, experiments were initiated at a cell density of $4 \times 10^5\ ml^{-1}$ (150 ml per point) to obtain an optimal cell harvest for the DNA extraction procedure. Comparisons between cellular, nuclei or DNA-associated platinum were carried out using Welch's alternative *t*-test, owing to the variances between standard deviations and two-tailed *P*-values calculated.

### Results

### *Growth-delay assays*

$IC_{50}$ values for all of the platinum(IV) dicarboxylates against the L1210/S and platinum-resistant variants are shown in Tables I–III. Comparing JM222 with JM223 (Table I), in which the amine ligand was isobutyl and the axial ligands either acetato (JM222) or butyrato (JM223), potency increased about 10-fold with extending the chain length of the axial ligands. When the amine ligand was *n*-propyl and with aryl substituents on the axial ligands (JM244), the $IC_{50}$ value was reduced almost 200-fold. The most active compound in the alicyclic series of ammine/amine dicarboxylates (axial ligand = butyrato) was JM221 (R = cyclohexyl, Table II). Table III shows the results obtained with cyclohexylamine/ammines carrying various axial dicarboxylate ligands (aliphatic, branched-chain aliphatic and aromatic). As axial straight-chain aliphatic substituents were extended in a step-

**Table I** Aliphatic ammine/amine dicarboxylates

| | | | $IC_{50}$ (µM) | | |
|---|---|---|---|---|---|
| *Compound* | $R_1$ | *L1210/S* | *L1210/cis (10-fold)* | *L1210/tetra (34-fold)* | *L1210/carbo (14-fold)* |
| JM222 | $CH_3$ | 5.5 | 5.9 (1.07) | 4.5 (0.82) | – |
| JM223 | n-$C_3H_7$ | 0.4 | 0.4 (1.0) | 0.29 (0.73) | 0.28 (0.7) |
| JM244 |  | 0.03 | 0.03 (1.0) | 0.03 (1.0) | 0.04 (1.3) |

The results are the mean of two separate experiments (triplicate cultures per point in each experiment). Figures in parentheses represent fold resistance. L1210/S, sensitive cell line; L1210/cis, cisplatin resistant; L1210/tetra, tetraplatin resistant; L1210/carbo, carboplatin resistant.

wise manner, so the potency of the compounds increased, e.g. comparing JM216, JM231 and JM221 the butyrato axial ligand (JM221) was the most effective. Branched-chain aliphatics were less effective in producing growth delays compared with their straight-chain counterparts. Overall, the most active compounds in Table III were those with aromatic axial ligands (JM2644 and JM290). The $IC_{50}$ values of JM2644 and JM290 were similar to the $IC_{50}$ value of JM244 (aromatic axial ligands, aliphatic amine ligand) in Table I. All of the platinum(IV) ammine/amine dicarboxylates overcame cisplatin, tetraplatin and carboplatin resistance in these L1210 lines, resistance factors being in the range of 0.4–2. JM221 was selected for further comparative studies with cisplatin as the most potent of the alicyclic compounds in Table II.

### *Cell survival*

Cell survival, as measured by colony-forming ability in soft agar following a 2 h exposure to varying concentrations of cisplatin, in the L1210/S and L1210/cis lines has been reported elsewhere (Nicolson *et al.*, 1992). In order to assess the biochemical parameters associated with longer term exposure to cisplatin or JM221 in the L1210/S and L1210/cis lines, cell survival experiments were carried out following 24 h exposure to either agent (Figure 1). The concentrations of compounds that resulted in 25% clonogenic survival were 0.6 μM cisplatin and 0.1 μM JM221 for L1210/S and 4.2 μM cisplatin and 0.15 μM JM221 for L1210/cis. The first three drug treatments were selected for further biochemical studies. At these drug concentrations cells continued to cycle through one doubling during the 24 h continuous exposure, and viabilities, as assessed by trypan blue dye exclusion, were >90%. JM221 treatment of the L1210/cis line was omitted owing to an apparent lack of cross-resistance in colony assays.

### *Biochemistry*

Following exposure of L1210/S and L1210/cis cells to varying concentratons of cisplatin (up to 100 μM) for 2 h, the L1210/cis line had approximately 50% less platinum associated with the DNA than the sensitive line at all drug concentrations (Figure 2). Viability studies (trypan blue dye exclusion) showed both cell lines to be intact following 2 h exposure to 100 μM cisplatin. At 10 μM cisplatin for 2 h, cell survival in clonogenic assays was 4% and 76% for L1210/S and L1210/cis respectively. These studies were extended to the measurement of cellular, nuclei and DNA platinum binding in cells exposed to either cisplatin or JM221 for 24 h at concentrations which resulted in 25% survival. L1210/cis cells exposed to a non-toxic concentration of cisplatin (0.6 μM) for 24 h were included for comparison with the sensitive line. At equimolar concentrations of cisplatin (0.6 μM) significantly less platinum was associated with the cells and DNA of the L1210/cis line compared with L1210/S (Table IV), whereas no statistical difference was shown with nuclei and DNA binding, although the means were lower. However, at equitoxic concentrations of cisplatin (L1210/S, 0.6 μM; L1210/cis, 4.2 μM) considerably more cell-associated

**Table II** Alicyclic ammine/amine dicarboxylates

| Compound | $R_1$ | *L1210/S* | *IC₅₀ (μM)* *L1210/cis (10-fold)* | *L1210/tetra (34-fold)* | *L1210/carbo (14-fold)* |
|---|---|---|---|---|---|
| JM260 | $c\text{-}C_4H_7$ | 0.18 | 0.22 (1.2) | 0.20 (1.1) | 0.26 (2.2) |
| JM229 | $c\text{-}C_5H_9$ | 0.25 | 0.25 (1.0) | 0.15 (0.6) | 0.25 (1.0) |
| JM221 | $c\text{-}C_6H_{11}$ | 0.06 | 0.11 (1.8) | 0.06 (1.0) | 0.12 (2.0) |
| JM271 | $c\text{-}C_7H_{13}$ | 0.12 | 0.15 (1.3) | 0.11 (0.9) | 0.26 (2.2) |

The results are the mean of two separate experiments (triplicate cultures per point in each experiment). Figures in parentheses represent fold resistance. L1210/S, sensitive cell line; L1210/cis, cisplatin resistant; L1210/tetra, tetraplatin resistant; L1210/carbo, carboplatin resistant.

**Table III** Cyclohexylamine dicarboxylates

| Compound | $R_1$ | *L1210/S* | *IC₅₀ (μM)* *L1210/cis (10-fold)* | *L1210/tetra (34-fold)* | *L1210/carbo (14-fold)* |
|---|---|---|---|---|---|
| JM216 | $CH_3$ | 1.2 | 1.5 (1.3) | 1.4 (1.2) | 1.6 (1.3) |
| JM231 | $C_2H_5$ | 0.31 | 0.35 (1.1) | 0.18 (0.6) | 0.39 (1.3) |
| JM221 | $n\text{-}C_3H_7$ | 0.06 | 0.11 (1.8) | 0.06 (1.0) | 0.12 (2.0) |
| JM272 | $i\text{-}C_3H_7$ | 0.14 | 0.1 (0.7) | 0.07 (0.5) | 0.21 (1.5) |
| JM262 | $t\text{-}C_3H_7$ | 0.18 | 0.15 (0.8) | 0.08 (0.4) | 0.15 (0.8) |
| JM274 | $n\text{-}C_4H_9$ | 0.05 | 0.08 (1.6) | 0.05 (1.0) | 0.08 (1.6) |
| JM273 | $t\text{-}C_4H_9$ | 0.08 | 0.07 (0.9) | 0.04 (0.5) | 0.11 (1.4) |
| JM256 | $NHC_2H_5$ | 1.27 | 1.38 (1.1) | 0.79 (0.6) | 1.58 (1.2) |
| JM321 | $NHC_4H_9$ | 0.06 | 0.07 (1.2) | 0.04 (0.7) | 0.10 (1.7) |
| JM2644 | Ph | 0.03 | 0.03 (1.0) | 0.03 (0.7) | 0.03 (1.0) |
| JM290 | $Ph\text{-}NO_2$ | 0.04 | 0.05 (1.3) | 0.04 (1.0) | 0.05 (1.3) |

The results are the mean of two separate experiments (triplicate cultures per point in each experiment). Figures in parentheses represent fold resistance. L1210/S, sensitive cell line; L1210/cis, cisplatin resistant; L1210/tetra, tetraplatin resistant; L1210/carbo, carboplatin resistant.

**Figure 1** Cytotoxicity of cisplatin to L1210/S (●—●) or L1210/cis (■—■) and JM221 to L1210/S (▲—▲) or L1210/cis (□—□), in soft-agar colony assays following 24 h continuous exposure. Each point represents the mean of four separate observations. Error bars representing ± s.d. are shown where they exceed the size of the symbols.

**Figure 2** Platinum binding to the DNA of L1210/S (●—●) and L1210/cis (■—■) cells following 2 h exposure to varying concentrations of cisplatin. The results are the means of two separate experiments. Error bars represent ± s.d.

**Table IV** Platinum associated with cells, nuclei and DNA of L1210 cells exposed to equitoxic or equimolar concentrations of cisplatin (L1210/S L1210/cis) or equitoxic concentrations of JM221 (L1210/S)

| Treatment | *nmoles of Pt/g DNA* Cells | Nuclei | DNA |
|---|---|---|---|
| A. L1210/S + 0.6 µM cisplatin[a] | 554 (± 16.2) | 110 (± 19.1) | 11.5 (± 1.8) |
| B. L1210/S + 0.1 µM JM221[a] | 2684 (± 572) | 154 (± 39.5) | 17.1 (± 1.6) |
| C. L1210/cis + 0.6 µM cisplatin | 246 (± 25.0) | 75 (± 25.5) | 7.5 (± 1.2) |
| D. L1210/cis + 4.2 µM cisplatin[a] | 2140 (± 538) | 272 (± 71.1) | 21.1 (± 1.4) |

[a]The concentration of compound leading to 25% cell survival following 24 h continuous exposure (see Figure 1). The results represent the mean (± s.d.) of three separate experiments. Comparing A with B, cells $P < 0.05$, nuclei $P > 0.05$, DNA $P < 0.05$; A with C, cells $P < 0.001$, nuclei $P > 0.05$, DNA $P > 0.05$; A with D, cells $P < 0.05$, nuclei $P > 0.05$; DNA $P < 0.01$; B with C, cells $P < 0.05$, nuclei $P > 0.05$, DNA $P < 0.01$; B with D, cells $P > 0.5$, nuclei $P > 0.05$, DNA $P > 0.05$; C with D, cells $P < 0.05$, nuclei $P < 0.05$, DNA $P < 0.01$ using Welch's alternative $t$-test as described in Materials and methods.

platinum (nearly 4-fold) and twice as much platinum was associated with the nuclei and DNA of the L1210/cis line. When the sensitive line was exposed to equitoxic concentrations of either cisplatin or JM221, it was evident that nearly five times more platinum was associated with whole cells following JM221 exposure, even though the added drug concentration was 6-fold lower than cisplatin (Table IV). Although more DNA platination (1.5-fold) was observed after exposure to JM221 compared with cisplatin, there was no significant difference between nuclear platinum concentrations.

### Discussion

Recently we showed that, although four series of novel ammine/amine platinum(II) and platinum(IV) complexes, broadly based on the structures of cisplatin, carboplatin, tetraplatin and iproplatin, can overcome acquired resistance to cisplatin in L1210 cells, the only class of compounds which can overcome acquired resistance to tetraplatin is the series of ammine/amine *trans*-dihydroxodichloroplatinum(IV) complexes (Orr *et al.*, 1993). Since this line is also sensitive to the *trans*-dihydroxodichloroplatinum(IV) parent diammine, it is apparent that the *trans*-dihydroxodichloro- ligand arrangement alone confers sensitivity, whereas the nature of the amine ligand determines potency. Now we are reporting a novel class of ammine/amine platinum(IV) dicarboxylates which can overcome acquired resistance to cisplatin, tetraplatin and carboplatin in three variant L1210 lines. With these dicarboxylates, it is apparent that the length and substituents of the axial ligands, as well as of the amine ligand, strongly influence potency. For example, extending a straight-chain aliphatic axial ligand from acetato to butyrato increases the potency 14-fold when these compounds possess a branched-chain amine ligand (comparing JM222 with JM223) and 20-fold when these compounds have an alicyclic amine ligand (comparing JM216 with JM221). In addition, the cyclohexylamine function confers greater potency on these compounds (4- to 7-fold) than the corresponding branched-chain aliphatic ligand (comparing JM216 with JM222 and JM221 with JM223). This is in agreement with the results of colleagues who have studied many of these dicarboxylates, including JM216, JM222 and JM221, using a panel of human ovarian cell lines with a broad spectrum of sensitivity to cisplatin (Kelland *et al.*, 1992). We also found that the most active compounds against the L1210 lines were those possessing aromatic axial ligands. These compounds were approximately twice as potent as JM221, regardless of whether the amine ligand was aliphatic (JM244) or alicyclic (JM2644). In cell survival experiments, following 24 h drug exposure to L1210/S cells, JM221 was found to be 6-fold more active than cisplatin, which prompted some preliminary comparative assessments of cellular accumulation and DNA binding.

Several studies have identified decreased intracellular platinum accumulation as one mechanism contributing to acquired resistance to cisplatin in L1210 cells (Hromas *et al.*, 1987; Richon *et al.*, 1987; Waud, 1987; Kraker & Moore, 1988). Recent characterisation of all of our resistant lines demonstrated reduced platinum uptake following exposure to cisplatin, tetraplatin or carboplatin, although this did not correlate with the degree of resistance (Nicolson *et al.*, 1992), while glutathione levels remained unchanged. These studies have been extended here to assess the degree of platinum binding to DNA in L1210/cis cells compared with L1210/S cells at equimolar and equitoxic concentrations of cisplatin. As expected, the cellular platinum content was significantly reduced in the L1210/cis line compared with the L1210/S line at equimolar concentrations of cisplatin following 24 h exposure, with lower amounts of platinum associated with nuclei and DNA, although the difference was not statistically significant. However, at equitoxic concentrations of cisplatin more platinum was associated with cells, nuclei and the extracted DNA from the resistant line than with the sensitive line, indicating a greater overall tolerance to platinum in L1210/cis cells. The L1210/cis cells had nearly four times more cellular platinum, leading to twice the amount of platinum bound to DNA, than the sensitive line at equitoxic concentrations of cisplatin. Therefore, reduced platinum uptake is only one feature of resistance in the L1210/cis line, which is probably multifactorial. At the DNA level, an additional mechanism may be an enhanced capacity for DNA repair, as others have documented using cisplatin-resistant L1210 cell lines developed elsewhere (Sheibani *et al.*, 1989). At the cellular level, cell volume, protein content and glutathione levels have not changed during the development of acquired resistance to cisplatin (Nicolson *et al.*, 1992; Orr *et al.*, 1993), and other workers have not found a role for elevated metallothioneins in an L1210 line with acquired resistance to cisplatin (Farnworth *et al.*, 1990).

When L1210/S cells were exposed to equitoxic concentrations of either cisplatin or JM221, nearly five times more platinum was associated with the JM221-treated cells even though the cells were exposed to a 6-fold lower concentration of JM221 than of cisplatin. This is probably because of the greater lipophilicity of JM221 (Giandomenico *et al.*, 1991), and this property may also contribute to the potency of some of these dicarboxylates. However, from the experiments reported here, we cannot determine whether the majority of the platinum is intracellular or merely bound on/in the cell membrane. Certainly, the amount of platinum bound to the DNA of JM221-treated cells (1.5-fold greater than the cisplatin-treated L1210/S cells) does not reflect the quantity of platinum associated with whole cells. *In vitro* binding studies of cisplatin or of JM221 at 10 $\mu$M to calf thymus DNA over 24 h at 37°C showed that six times more cisplatin was bound than JM221 (data not shown). Whether the parent compound or an intracellular platinum(II) or (IV) metabolite binds to the DNA in L1210 cells remains to be determined. Certainly, the L1210/tetra line is cross-resistant to the reduced platinum(II) metabolite JM118 [*cis*-amminedichloro (cyclohexylamine)platinum(II)], whereas sensitivity is retained with JM221. However, this cross-resistance may be at the level of cellular uptake of compound.

In summary, platinum(IV) dicarboxylates represent another class of platinum-containing agents which overcome acquired resistance to cisplatin, tetraplatin and carboplatin in L1210 cells. All of these lines exhibit reduced uptake of the parent compound. However, studies with the L1210/cis line here suggest that at least one other resistance mechanism exists, possibly at the DNA level. Comparing cisplatin with JM221 in the sensitive line at equitoxic concentrations, it is apparent that the high levels of cellular platinum achieved with JM221 contribute to the potency of this dicarboxylate.

This study was supported by grants to the Institute of Cancer Research from the Cancer Research Campaign and the Medical Research Council, the Johnson Matthey Technology Centre and Bristol Myers Squibb Oncology.

**References**

BRAMWELL, V.H.C., CROWTHER, D., O'MALLEY, S., SWINDELL, R., JOHNSON, R., COOPER, E.H., THATCHER, N. & HOWELL, A. (1985). Activity of JM-9 in advanced ovarian cancer; a phase I-II trial. *Cancer Treat. Rep.*, **69**, 409-416.

BURCHENAL, J.H., KALAHER, K., DEW, K. & LOKYS, L. (1979). Rationale for development of platinum analogues. *Cancer Treat. Rep.*, **63**, 1493-1498.

BURTON, K. (1956). A study of the conditions and mechanism of the diphenylamine reaction for the colorimetric estimation of deoxyribonucleic acid. *Biochem. J.*, **62**, 315-323.

FARNWORTH, P., HILLCOAT, B. & ROOS, I. (1990). Metallothionein-like proteins and cell resistance to cis-dichlorodiammineplatinum (II) in L1210 cells. *Cancer Chemother. Pharmacol.*, **25**, 411-417.

GIANDOMENICO, C.M., ABRAMS, M.J., MURRER, B.A., VOLLANO, J.F., HARRAP, K.R., GODDARD, P.M., KELLAND, L.R. & MORGAN, S.E. (1991). Synthesis and reactions of a new class of orally active Pt(IV) antitumour complexes. In *Proceedings of the Sixth International Symposium on Platinum and Other Metal Coordination Compounds in Cancer Chemotherapy*, Howell, S.B. (ed.) pp. 93-100. Plenum Press: New York.

GIBBONS, G.R., WYRICK, S. & CHANEY, S.G. (1989). Rapid reduction of tetrachloro(D,L-trans)1,2-diaminocyclohexaneplatinum (IV) (tetraplatin) in RPMI-1640 tissue culture medium. *Cancer Res.*, **49**, 1402-1407.

GOTTLIEB, J.A. & DREWINKO, B. (1975). Review of the current clinical status of platinum coordination complexes in cancer chemotherapy. *Cancer Chemother. Rep.*, **59**, 621-628.

HARRAP, K.R. (1985). Preclinical studies identifying carboplatin as a viable cisplatin alternative. *Cancer Treat. Rev.*, **12**, 21-33.

HROMAS, R.A., NORTH, J.A. & BURNS, C.P. (1987). Decreased cisplatin uptake by resistant L1210 leukaemia cells. *Cancer Lett.*, **36**, 197-201.

KELLAND, L.R., MURRER, B.A., ABEL, G., GIANDOMENICO, C.M. & MISTRY, P. (1992). Ammine/amine platinum(IV) dicarboxylates: a novel class of platinum complex exhibiting selective cytotoxicity to intrinsically cisplatin-resistant human ovarian carcinoma cell lines. *Cancer Res.*, **52**, 822-828.

KIRBY, K.S. (1965). Isolation and characterisation of ribosomal ribonucleic acid. *Biochem. J.*, **96**, 266-269.

KRAKER, A.J. & MOORE, C.W. (1988). Accumulation of cis-diamminedichloroplatinum(II) and platinum analogues by platinum-resistant murine leukaemia cells *in vitro*. *Cancer Res.*, **48**, 9-13.

KRAKOFF, I.H. (1979). Nephrotoxicity of cis-dichlorodiammine-platinum(II). *Cancer Treat. Rep.*, **63**, 1523-1525.

MANGIONI, C., BOLIS, G., PECORELLI, S., BRAGMAN, K., EPIS, A., FAVALLI, G., GAMBINO, A., LANDONI, F., PRESTI, M., TORRI, W., VASSENA, L., ZANABONI, F. & MARSONI, S. (1989). Randomised trial in advanced ovarian cancer comparing cisplatin and carboplatin. *J. Natl Cancer Inst.*, **81**, 1464-1471.

NICOLSON, M.C., ORR, R.M., O'NEILL, C.F. & HARRAP, K.R. (1992). The role of platinum uptake and glutathione levels in L1210 cells sensitive and resistant to cisplatin, tetraplatin or carboplatin. *Neoplasma*, **39**, 189-195.

ORR, R.M., BARNARD, C.F.J., MURRER, B.A., O'NEILL, C.F., NICOLSON, M.C., BALAZOVA, E. & HARRAP, K.R. (1993). Evaluation of novel platinum (II) and platinum (IV) ammine/amine complexes in L1210 murine leukaemia cell lines sensitive and resistant to cisplatin and tetraplatin. *Cell Pharmacol.*, **1**, 17-23.

OZOLS, R.F. & YOUNG, R.C. (1984). Chemotherapy of ovarian cancer. *Semin. Oncol.*, **11**, 251-263.

PENDYALA, L., COWENS, J.W., CHHEDA, G.B., DUTTA, S.P. & CREAVEN, P.J. (1988). Identification of cis-dichloro-bis-isopropylamine platinum(II) as a major metabolite of iproplatin in humans. *Cancer Res.*, **48**, 3533-3536.

PENDYALA, L., ARAKALI, A.V., SANSONE, P., COWENS, J.W. & CREAVEN, P.J. (1990). DNA binding of iproplatin and its divalent metabolite cis-dichloro-bis-isopropylamine platinum(II). *Cancer Chemother. Pharmacol.*, **27**, 248-250.

RICHON, V.M., SCHULTE, N. & EASTMAN, A. (1987). Multiple mechanisms of resistance to cis-diamminedichloroplatinum(II) in murine leukaemia L1210 cells. *Cancer Res.*, **47**, 2056-2061.

ROSE, W.C., SCHURIG, J.E., HUFTALEN, J.B. & BRADNER, W.T. (1982). Antitumour activity and toxicity of cisplatin analogs. *Cancer Treat. Rep.*, **66**, 135-146.

ROSENBERG, B. (1985). Fundamental studies with cisplatin. *Cancer*, **55**, 2303-2316.

SCHILDER, R.J., LACRETA, F.P., PEREZ, R.P., NASH, S., HAMILTON, T.C., GOLDSTEIN, L.J., YOUND, R.C., OZOLS, R.F. & O'DWYER, P.J. (1987). Phase I/Pharmacokinetic study of ormaplatin (tetraplatin, NSC363812) on a day 1 and 8 schedule (abstract). *Proc. Am. Assoc. Cancer Res.*, **33**, 537.

SCHMIDT, G. & THANNHAUSER, S.J. (1945). Detection of desoxyribonucleic, ribonucleic acid and phosphoproteins in animal tissues. *J. Biol. Chem.*, **161**, 83-89.

SHEIBANI, N., JENNERWEIN, M.M. & EASTMAN, A. (1989). DNA repair in cells sensitive and resistant to cis-diamminedichloroplatinum(II): host cell reactivation of damaged plasmid DNA. *Biochemistry*, **28**, 3120-3124.

WAUD, W.R. (1987). Differential uptake of cis-diamminedichloroplatinum(II) by sensitive and resistant L1210 leukaemia cells. *Cancer Res.*, **47**, 6549-6555.